# Serum Cholesterol and the Progression of Parkinson's Disease: Results from DATATOP

**DOI:** 10.1371/journal.pone.0022854

**Published:** 2011-08-11

**Authors:** Xuemei Huang, Peggy Auinger, Shirley Eberly, David Oakes, Michael Schwarzschild, Alberto Ascherio, Richard Mailman, Honglei Chen, for the Parkinson Study Group DATATOP Investigators

**Affiliations:** 1 Departments of Neurology, Neurosurgery, Pharmacology, Radiology, and Kinesiology, Pennsylvania State University-Milton Hershey Medical Center, Hershey, Pennsylvania, United States of America; 2 Department of Neurology, Center for Human Experimental Therapeutics, University of Rochester School of Medicine and Dentistry, Rochester, New York, United States of America; 3 Department of Biostatistics, University of Rochester School of Medicine and Dentistry, Rochester, New York, United States of America; 4 Department of Neurology, Massachusetts General Hospital, Boston, Massachusetts, United States of America; 5 Departments of Nutrition and Epidemiology, Harvard School of Public Health, Boston, Massachusetts, United States of America; 6 Departments of Neurology and Pharmacology, Pennsylvania State University-Milton Hershey Medical Center, Hershey, Pennsylvania, United States of America; 7 Epidemiology Branch, National Institute of Environmental Health Sciences, Research Triangle Park, North Carolina, United States of America; University of North Dakota, United States of America

## Abstract

**Background:**

Recent studies have suggested that higher serum cholesterol may be associated with lower occurrence of Parkinson's disease (PD). This study is to test the hypothesis that higher serum cholesterol correlates with slower PD progression.

**Methods:**

Baseline non-fasting serum total cholesterol was measured in 774 of the 800 subjects with early PD enrolled between 1987 and 1988 in the Deprenyl and Tocopherol Antioxidative Therapy of Parkinsonism (DATATOP) trial. Participants were followed for up to two years, with clinical disability requiring levodopa therapy as the primary endpoint. Hazard ratios (HRs) and 95% confidence intervals (CI) were determined for increasing serum cholesterol concentration (in quintiles) for clinical disability requiring levodopa therapy, after adjusting for confounders. At baseline, only nine subjects reported use of cholesterol-lowering agents (two with statins).

**Results:**

The overall mean cholesterol level was 216 mg/dL (range 100–355). The HR of progressing to the primary endpoint decreased with increasing serum cholesterol concentrations. Compared to the lowest quintile, the HRs (*95%CI*), for each higher quintile (in ascending order) are 0.83 *(0.59–1.16)*; 0.86 *(0.61–1.20)*; 0.84 *(0.60–1.18)*; and 0.75 *(0.52–1.09)*. The HR for one standard deviation (SD) increase = 0.90 [(0.80–1.01), p for trend = 0.09]. This trend was found in males (HR per SD = 0.88 [(0.77–1.00), p for trend = 0.05], but not in females [HR = 1.03 (0.81–1.32)].

**Conclusions:**

This secondary analysis of the DATATOP trial provides preliminary evidence that higher total serum cholesterol concentrations may be associated with a modest slower clinical progression of PD, and this preliminary finding needs confirmation from larger prospective studies.

## Introduction

As an age-related neurodegenerative disorder, Parkinson's disease (PD) affects about one million Americans, and these numbers are expected to grow as the population ages. Although the etiology is known in a small percentage of genetically related cases, the disorder is largely idiopathic, and likely involves interactions of the genome and the environment [1]. Three recent case-control studies [2]–[4] suggest that higher serum cholesterol levels may be related to lower prevalence of PD. Three independent prospective studies provided further support for the hypothesis that higher serum cholesterol may be associated with a lower future risk of PD [5]–[7]. A fourth prospective study whose PD case identification was based on a Finnish National Insurance Register that entitles patients to medication free of charge, however, offered a contradictory finding [8]. Although the findings are still controversial, the overall evidence favors an association between higher cholesterol and lower PD occurrence, suggesting a beneficial role of higher serum cholesterol in the PD process. No study, however, has examined the relationship between serum cholesterol and PD progression. In this study, we tested the hypothesis that higher serum cholesterol levels may be associated with slower progression of PD by analyzing the data collected from the Deprenyl and Tocopherol Antioxidative Therapy of Parkinsonism (DATATOP) trial.

## Methods

### Study Design

The DATATOP study is a two-year, double-blind, randomized trial originally designed to test the hypothesis that long-term treatment of early PD with the monoamine oxidase type B inhibitor deprenyl (selegiline hydrochloride) and/or the antioxidant α-tocopherol would extend the time until the emergence of disability requiring therapy with levodopa. Cholesterol profiles were collected for 774 of the 800 study participants at enrollment (1987–1988). The full list of DATATOP investigators/coordinators can be found in the file “Supporting Information S1.” The data from DATATOP is suitable for this initial exploration of our hypothesis because the study started in the late 1980's prior to aggressive campaigns to lower cholesterol using drugs like statins. Thus, baseline cholesterol levels collected in DATATOP may better reflect the pre-morbid long-term exposure to plasma cholesterol that is key for testing our hypothesis.

### Study Subjects

Eight hundred PD subjects without severe postural instability, within five years of symptoms onset, and not yet requiring symptomatic therapy, were enrolled in the DATATOP study between September 1987 and November 1988. Subjects enrolled in the study had mild PD symptoms with Hoehn and Yahr stages between 1 and 2. Patients were excluded if they had severe tremor, serious dementia (Mini-Mental State Examination (MMSE) score ≤22), or depression (Hamilton Scale for Depression score ≥16). Subjects were evaluated and examined by PD specialists. After baseline evaluation, study participants were randomized according to a 2×2 factorial design to one of four treatment assignments: deprenyl (10 mg/d) and α-tocopherol placebo, α-tocopherol (2000 IU/d) and deprenyl placebo, active deprenyl and active α-tocopherol, or double placebo [9]. The original DATATOP study had received IRB approval from all of the participating sites, and all subjects gave informed consent. The current study used de-identified data, and required neither IRB approval, nor informed consent.

### Determination of serum total cholesterol levels

Non-fasting blood specimens were obtained at the baseline visit and every 6 months as part of the routine laboratory surveillance. Blood was allowed to clot for 30 minutes and then centrifuged within 1 hour after collection until clot and serum were separated by a well-formed polymer barrier. The serum was then transferred into a plastic vial, and sent at ambient temperature to a centralized laboratory. This laboratory determined total cholesterol concentrations as part of the routine serum chemistry profile to allow detection of any abnormalities that might have developed during the study. Ideally, we would have liked fasting cholesterol profiles, yet Weiss and Colleagues compared the non-fasting and fasting cholesterol profiles of 100 consecutive patients with and without diabetes [10], and found no significant difference between non-fasting and fasting total cholesterol levels. Nevertheless, future studies with lipid profiles measured in fasting state and cholesterol being fractioned as HDL and LDL would be useful.

### Ascertainment of the primary endpoint

Following the baseline visit and initiation of study drugs, subjects were scheduled for visits every three months until 24 months had elapsed [9]. At each visit the site investigator evaluated the subject for disability sufficient to require dopaminergic therapy, which was defined as the primary end point for this study. Of the 774 subjects with baseline cholesterol levels, 369 reached this primary end point during the study period.

### Ascertainments of secondary endpoints

At each visit, the Unified Parkinson's Disease Rating Scale (UPDRS) score (sum of the motor, cognitive, and activity of daily living subscale scores) was obtained [9]. A secondary endpoint for this study was the annualized rate of change in the UPDRS scores from baseline to the primary endpoint (or the final visit if the primary end point was not reached) for each subject and was calculated as follows: *ΔUPDRS = (UPDRS1-UPDRS2)/T/365*; where *ΔUPDRS* = annualized rate of change in the UPDRS, *UPDRS_2_* = total UPDRS score at the last assessment before initiation of dopaminergic treatment, *UPDRS_1_* = total UPDRS score at baseline; and T = number of days between the two assessments.

The vital status and date of death of participants in the DATATOP trial were updated in 2001 to 2002 as previously described [11]. Another secondary endpoint for this study was the time until death as determined at the time of this update. The shortest time elapsed between enrollment and vital status update was 13 years. At the time of the update, 287 of the 774 subjects for whom there were baseline serum cholesterol data were identified as deceased.

Freezing of gait (FOG) was determined from the activities of daily living (ADL) section of the UPDRS and was considered to be present if the score was ≥1 on the FOG question. An additional secondary outcome measurement for this study was the time from randomization until FOG score on the UPDRS became ≥1 as previously described [12]. There were 57 patients (7.4%) who experienced FOG by the time of the baseline evaluation and were excluded from analyses for this outcome. Of the remaining 717 patients, 147 reached the endpoint of FOG.

### Statistical analysis

Baseline characteristics of the study participants by baseline serum cholesterol quintiles are reported as mean or median values for continuous measures and percentages for categorical measures. ANOVAs, Wilcoxon scores, and Chi-square statistics tested for statistically significant differences among the cholesterol quintiles. Tests for linear trend across the cholesterol quintiles were assessed by including cholesterol quintiles as a continuous variable in the linear models for continuous measures and by the Cochran-Armitage trend test for categorical measures.

Cox proportional hazards models were used to estimate the Hazard Ratios (HRs) and 95% confidence interval (CI) of reaching the primary end point according to quintiles of baseline serum total cholesterol concentration after adjusting for confounders. Confounders included gender, treatment group (deprenyl or not), baseline age, uric acid concentration [13], PD subtype (tremor, postural instability gait disorder (PIGD), or mixed) [14], and body mass index (BMI). Models that further adjusted for baseline reported use of antihypertensive medications, non-steroidal anti-inflammatory drugs (NSAIDs), and smoking status did not appreciably alter the results and therefore detailed results from this additional analysis are not presented. Initial analyses were conducted using quintiles based on the combined total cholesterol concentration distribution in both males and females. Secondary gender specific analyses were conducted based on gender-specific quintiles because overall women have a lower PD incidence [1] and also a different pattern of age-related serum cholesterol changes compared to men [15]. Tests for linear trend were conducted by including serum cholesterol concentration as a continuous variable in the proportional hazards models. Similar analyses also were conducted for secondary endpoints: time to death and time to FOG.

The relationship between serum cholesterol concentration and rate of change in the UPDRS score in the total cohort was assessed by linear regression with model adjustment noted above as well as adjusting for baseline UPDRS score. Adjusted means for annualized rate of change in UPDRS scores are reported.

Potential interactions between cholesterol levels and gender, as well as with treatment groups, were explored by including the cross-product of serum cholesterol level as a continuous variable with deprenyl (yes/no) and α-tocopherol (yes/no) in the proportional hazards and linear regression models. No significant interactions were observed for any of the four outcome measures and thus results for these models are not presented further.

## Results

The overall mean cholesterol level was 216 mg/dL (range 100–355) (Table 1). Participants in the higher cholesterol quintiles were more likely to be female (p<0.001). Population characteristics were otherwise similar across quintiles of baseline cholesterol concentrations. Of all patients, only nine reported the use of cholesterol-lowering medications at baseline, and two of them indicated statin use. The cholesterol levels did not vary significantly by treatment group (deprenyl = 217.6 mg/dL, α-tocopherol = 213.3 mg/dL, both = 213.1 mg/dL, placebo = 214.3 mg/dL, p = 0.64).

**Table 1:** Baseline characteristics of study participants according to quintiles of baseline serum cholesterol concentration.

	Baseline Serum Cholesterol quintiles						P values	
Characteristic	1^st^	2^nd^	3^rd^	4^th^	5^th^	Overall	Overall	Trend
Serum cholesterol concentration (mg/dL)	≤180.7	180.8–203.9	204.0–222.8	222.9–246.7	≥246.8	216.2^a^		
Subjects, No.	153	159	153	156	153	774		
Female, %	27.5	28.3	30.1	39.1	45.8	34.1	0.002	<0.001
Age, median, y	62	62	62	62	63	62	0.56	0.02
Body Mass Index (BMI), mean	25.8	26.0	26.2	25.6	26.7	26.1	0.16	0.20
Current smokers, %	11.8	7.8	7.2	7.7	9.8	8.8	0.57	0.61
Baseline medication use, %								
Any antihypertensive meds	22.2	30.2	27.5	24.4	28.1	26.5	0.53	0.61
Any NSAIDs	22.9	29.6	25.5	30.1	25.5	26.7	0.56	0.61
Any cholesterol lowering meds	1.3	0.0	1.3	0.6	2.6	1.2	0.28	0.23
Statins	0.0	0.0	0.7	0.0	0.7	0.3	0.55	0.31
Non statins	1.3	0.0	0.7	0.6	2.0	0.9	0.43	0.42
Caffeine containing meds	1.3	1.3	2.6	1.3	1.3	1.6	0.84	0.99
Cardiac comorbidity, %	21.6	28.9	31.4	23.1	26.8	26.4	0.27	0.69
Time since onset per rater, mean, y	2.2	2.2	2.2	2.1	1.9	2.1	0.26	0.10
Total UPDRS score, mean	27.0	25.1	25.5	25.9	23.8	25.4	0.21	0.06
UPDRS tremor score, mean	4.7	4.5	4.7	4.6	4.4	4.6	0.87	0.57
MMSE score, mean	28.8	28.9	28.9	28.7	28.9	28.8	0.75	0.84
Serum urate concentration, mean, mg/dL	5.0	4.9	5.1	5.0	5.3	5.1	0.09	0.05
PD subtype (tremor/PIGD ratio), %							0.96	
Tremor predominant (ratio ≥1.5)	54.2	54.1	53.6	54.5	56.8	54.6		0.66
PIGD predominant (ratio ≤1.0)	28.8	27.0	31.4	31.4	28.8	29.5		0.70
Mixed (ratio 1.0–1.5)	17.0	18.9	15.0	14.1	14.4	15.9		0.28

NSAID = non-steroidal anti-inflammatory drug; UPDRS = Unified Parkinson's Disease Rating Scale; MMSE = Mini-Mental State Examination; PIGD = postural instability gait disorder.

a Value is expressed as the mean.

Higher cholesterol levels tended to be associated with lower risk of reaching the primary endpoint with a borderline statistical significance (Tale 2, p for trend = 0.09). Compared to the lowest quintile, the HRs (*95% CI*), for each higher quintile (in ascending order) are 0.83 *(0.59–1.16)*; 0.86 *(0.61–1.20)*; 0.84 *(0.60–1.18)*; and 0.75 *(0.52–1.09)* respectively. The HR for each standard deviation (SD) increase = 0.90 (0.80–1.01, p = 0.09). The subgroup analysis in males showed a statistical difference between the highest cholesterol quintile and the lowest in reaching the primary endpoint [HR = 0.59 (0.38–0.93), p = 0.02]. The HR for each 1 SD increase in males was 0.88 [(0.77–1.00), p = 0.05]. No such relationship was seen in females (Table 2).

**Table 2:** Hazard ratios for reaching the primary end point according to quintiles of baseline serum cholesterol concentration or corresponding to a 1-SD increase in serum cholesterol concentration.

			Need dopaminergic therapy	
Serum cholesterol concentration quintile	Serum cholesterol concentration (mg/dL)	No. of subjects	HR (95% CI)	Two-tailp value
*All subjects*				
1^st^	≤180.7	153	1.0 (Reference)	–
2^nd^	180.8–203.9	159	0.82 (0.59–1.16)	0.26
3^rd^	204.0–222.8	153	0.86 (0.62–1.20)	0.36
4^th^	222.9–246.7	156	0.85 (0.60–1.19)	0.33
5^th^	≥246.8	153	0.75 (0.52–1.09)	0.13
1-SD^a^ increase n serum cholesterol concentration			0.91 (0.81–1.02)	0.09
*Males*				
1^st^	≤178.8	101	1.0 (Reference)	–
2^nd^	178.9–198.8	103	0.79 (0.52–1.20)	0.26
3^rd^	198.9–216.6	104	0.98 (0.66–1.46)	0.93
4^th^	216.7–241.8	103	1.06 (0.72–1.57)	0.76
5^th^	≥241.8	99	0.59 (0.38–0.93)	0.02*
1-SD increase in serum cholesterol concentration			0.88 (0.77–1.001)	0.05
*Females*				
1^st^	≤185.7	53	1.0 (Reference)	–
2^nd^	185.8–211.6	54	0.77 (0.39–1.51)	0.44
3^rd^	211.7–233.6	51	0.63 (0.30–1.31)	0.21
4^th^	233.7–255.6	53	0.87 (0.43–1.76)	0.70
5^th^	≥255.7	53	1.06 (0.51–2.21)	0.87
1-SD increase in serum cholesterol concentration			1.03 (0.81–1.32)	0.81

HR = hazard ratio; CI = confidence interval. Models are adjusted for gender, treatment group, baseline age, uric acid concentration, PD subtype, and BMI.

a A 1-standard deviation (SD) increase indicates an increase of 39.2 mg/dL in all subjects, 37.6 mg/dL in males, and, 41.0 mg/dL in females.

The annual rates of change in UPDRS scores are highest in the PD subjects with the lowest quintile of baseline total serum cholesterol (Table 3), but there were no statistically significant differences in any group. Similarly, the results in Table 4 show no associations between baseline total cholesterol and either the time to death or the time to freezing of gait.

**Table 3:** Adjusted means for annualized rate of change in UPDRS scores according to quintiles of baseline serum cholesterol concentration or corresponding to a 1-SD increase in serum cholesterol concentration.

			Annualized rate of change in UPDRS scores	
Serum cholesterol concentration quintile	Serum cholesterol concentration, mg/dL	No.	Adjusted mean(95% CI)	Two tailp-value
*All subjects*				
1^st^	≤180.7	153	14.68 (10.58–18.78)	–
2^nd^	180.8–203.9	159	10.78 (6.72–14.85)	0.13
3^rd^	204.0–222.8	153	12.34 (8.08–16.59)	0.36
4^th^	222.9–246.7	156	11.55 (7.33–15.78)	0.23
5^th^	≥246.8	153	12.76 (8.48–17.05)	0.47
1-SD^a^ increase in serum cholesterol concentration			−0.61 (−2.25–1.03)	0.47
*Males*				
1^st^	≤178.8	101	14.26 (9.51–19.01)	–
2^nd^	178.9–198.8	103	12.75 (7.93–17.56)	0.61
3^rd^	198.9–216.6	104	15.01 (10.15–19.88)	0.79
4^th^	216.7–241.8	103	13.03 (8.28–17.79)	0.67
5^th^	≥241.8	99	12.65 (7.68–17.62)	0.59
1-SD increase in serum cholesterol concentration			−0.49 (−2.32–1.34)	0.60
*Females*				
1^st^	≤185.7	53	15.37 (7.63–23.12)	–
2^nd^	185.8–211.6	54	6.46 (−1.60–14.52)	0.08
3^rd^	211.7–233.6	51	4.98 (−3.56–13.53)	0.05
4^th^	233.7–255.6	53	10.46 (1.81–19.10)	0.37
5^th^	≥255.7	53	12.15 (3.48–20.82)	0.56
1-SD increase in serum cholesterol concentration			−0.51 (−3.97–2.96)	0.77

CI = confidence interval. Models are adjusted for gender, treatment group, baseline UPDRS score, age, uric acid concentration, PD subtype, and BMI.

a A 1-standard deviation (SD) increase indicates an increase of 39.2 mg/dL in all subjects, 37.6 mg/dL in males, and, 41.0 mg/dL in females.

**Table 4:** Hazard ratios for reaching secondary end points according to quintiles of baseline serum cholesterol concentration and the corresponding to a 1-SD increase in serum cholesterol concentration.

			Secondary Endpoint			
			Time to Death		Time to Freezing of gait	
Cholesterol conc. quintile	Cholesterol conc. (mM)	No. of subjects	HR (95% CI)	Two-tailp-value	HR (95% CI)	Two-tailp-value
1^st^	≤180.7	153	1.0 (Reference)	–	1.0 (Reference)	–
2^nd^	180.8–203.9	159	1.09 (0.75–1.59)	0.65	0.73 (0.42–1.28)	0.27
3^rd^	204.0–222.8	153	1.10 (0.75–1.62)	0.63	1.03 (0.61–1.76)	0.90
4^th^	222.9–246.7	156	1.16 (0.78–1.72)	0.46	0.83 (0.48–1.44)	0.51
5^th^	≥246.8	153	1.01 (0.68–1.50)	0.96	0.91 (0.52–1.58)	0.73
1 SD^a^ increase in serum cholesterol concentration			1.01 (0.90–1.15)	0.84	0.97 (0.81–1.16)	0.72
*Males*						
1^st^	≤178.8	101	1.0 (Reference)	–	1.0 (Reference)	–
2^nd^	178.9–198.8	103	1.10 (0.71–1.70)	0.67	0.61 (0.30–1.25)	0.18
3^rd^	198.9–216.6	104	1.16 (0.73–1.83)	0.53	1.18 (0.63–2.20)	0.61
4^th^	216.7–241.8	103	1.19 (0.76–1.87)	0.45	0.76 (0.39–1.50)	0.43
5^th^	≥241.8	99	0.89 (0.55–1.45)	0.64	0.88 (0.46–1.70)	0.71
1-SD increase in serum cholesterol concentration			1.00 (0.87–1.15)	0.99	0.93 (0.76–1.15)	0.52
*Females*						
1^st^	≤185.7	53	1.0 (Reference)	–	1.0 (Reference)	–
2^nd^	185.8–211.6	54	0.77 (0.30–1.98)	0.59	0.45 (0.15–1.34)	0.15
3^rd^	211.7–233.6	51	1.19 (0.52–2.72)	0.69	0.39 (0.12–1.25)	0.11
4^th^	233.7–255.6	53	0.86 (0.36–2.03)	0.73	0.98 (0.36–2.71)	0.97
5^th^	≥255.7	53	1.26 (0.55–2.90)	0.59	0.57 (0.19–1.71)	0.31
1-SD increase in serum cholesterol concentration			1.12 (0.86–1.47)	0.40	0.97 (0.67–1.39)	0.85

HR = hazard ratio; CI = confidence interval. Models are adjusted for gender (for total cohort), treatment group, baseline age, uric acid concentration, PD subtype, and BMI.

a A 1-standard deviation (SD) increase indicates an increase of 39.2 mg/dL in all subjects, 37.6 mg/dL in males, and, 41.0 mg/dL in females.

## Discussion

Prior literature has suggested that lower cholesterol may be associated with higher risk of PD [2]–[7], but there have been no studies to determine if there also is an association with faster PD progression. The statistical analysis of the relationship between baseline serum cholesterol and PD progression, was, however, borderline if based on p values of two tail t-test (p = 0.09 for whole cohort, 0.05 for male cohort). If one, however, assumes that a unidirectional outcome was predicted from the prior clinical data [2]–[7] and the study was to provide pilot data for pursuing future studies to test the hypothesis, a one-tailed analysis is justified [16] and makes the results significant for the group as a whole (p = 0.04), as well as for just males (p = 0.02). This study provides the first preliminary evidence that lower total serum cholesterol also may be associated with modestly faster progression of PD symptoms, supported the need for further investigation into the relationship between cholesterol and PD progression.

The question of whether low cholesterol contributes to faster PD progression, is merely a marker of more advanced pathology of PD, or is simply an epiphenomenon, cannot be addressed from this study. Cholesterol is involved in a plethora of critical biological functions ranging from cellular repair or degeneration [17]–[22] to being a neurosteroid precursor [23]–[26]. Whereas cholesterol may affect PD etiology or clinical progression, it is also conceivable that lower cholesterol could be a mere marker for a more advanced pathology of PD. Thus, understanding the nature and underlying mechanisms of the associations between lower cholesterol and increased PD risk or faster progression may have a profound influence in understanding key aspects of sporadic PD. Intriguingly, potential beneficial roles of higher cholesterol in other neurodegenerative disorders also have been implied. Hyperlipidemia was recently found to be a significant prognostic factor for survival of patients with amytrophic lateral sclerosis (ALS) [27], and lower cholesterol has also been suggested to be related with multisystem atrophy [28]. While this recent evidence is preliminary, it underscores the importance of understanding the role of cholesterol in neurodegenerative diseases. If the association turns out to be causal, there may be public health implications because aggressively lowering cholesterol has been advocated based on possible cardiovascular benefits.

An important strength of this current study is that it was initiated with primary endpoints evaluated mostly in the pre-statin era with less than 0.3% of the participants reporting statin usage at baseline. Statins are now commonly and sometimes aggressively used to lower serum cholesterol for cardiovascular benefits [29], [30]. Although the epidemiological data are preliminary and inconsistent, statin use has been hypothesized to be neuroprotective against PD [31]–[33]. Because statins effectively decrease plasma cholesterol and because statins may modify PD pathogenesis, it is difficult to determine the independent effect of chronic levels of plasma cholesterol on PD progression among populations with prevalent statin use. Another dataset of potential interest was the PRECEPT study, a neuroprotection trial conducted by PSG investigators that began in 2002 [34]. In PRECEPT, there were significant increases of statin usage with each lower quintile of total serum cholesterol levels (i.e., the percentage of the PD subjects reported statin usage from lowest to highest quintiles was: 37.6%, 26.4%, 19.4%, 10.8%, and 6.8% respectively). No significant association (unpublished data) was found between baseline cholesterol levels and PD progression (i.e., time to need dopaminergic therapy), but the analysis is confounded by the high degree of use of cholesterol-lowering medication (i.e., the measured cholesterol levels do not reflect premorbid levels). The fact that, in the current study, our primary end point was evaluated before the widespread use of statins largely eliminates these potential concerns.

In addition to sample size, the current study has other limitations. Total cholesterol was not measured in the fasting condition, and cholesterol was not fractioned as HDL and LDL-components. Moreover, and as noted earlier, the limited sample size particularly impacts the FOG analysis. Nonetheless, this is the first suggestion that there is an association of low cholesterol and faster PD progression, and is consistent with prior reports of an association with higher risk of PD. Since these measures are often part of routine clinical assessment, it may be that future studies in large groups of PD patients should include a fasting cholesterol measurement, and incorporate this and the use of statins and other cholesterol lowering drugs into data analyses. If these preliminary findings are supported, than basic studies of the potential underlying mechanism(s) would be warranted.

## Supporting Information

Supporting Information S1 This file contains the names and roles of the many individuals that designed, conducted, and did initial analyses of the DATATOP study.
(DOC)
