# Supplementary material for: Serum Cholesterol and the Progression of Parkinson's Disease: Results from DATATOP
Source: PLoS One. 2011 Aug 11;6(8):e22854. doi: 10.1371/journal.pone.0022854 (PMC3154909; doi:10.1371/journal.pone.0022854)
Supplement: Supporting Information S1 — This file contains the names and roles of the many individuals that designed, conducted, and did initial analyses of the DATATOP study. (DOC) [file pone.0022854.s001.doc]

The following are Contributors to the DATATOP trial:

(From: Parkinson Study Group. Effects of tocopherol and deprenyl on the progression of disability in early Parkinson's disease. N Engl J Med. 1993; 328:176-183):

Participating Investigators -- W. Koller, University of Kansas, Kansas City; C.W. Olanow, University of South Florida, Tampa; R. Rodnitzky, University of Iowa, Iowa City; J.S. Fink and J.H. Growdon, Massachusetts General Hospital, Boston; G. Paulson, Ohio State University, Columbus; R. Kurlan, University of Rochester, Rochester, N.Y.; J.H. Friedman, Roger Williams General Hospital, Providence, R.I.; S. Gancher and J. Nutt, Oregon Health Sciences University, Portland; A.H. Rajput, University of Saskatchewan, Saskatoon; J.B. Bennett and G.F. Wooten, University of Virginia, Charlottesville; P. LeWitt, Sinai Hospital, Detroit; C. Goetz, C. Tanner, and K. Shannon, Rush-Presbyterian-St. Luke's Medical Center, Chicago; O. Suchowersky, University of Calgary, Calgary, Alta.; M.F. Brin and S.B. Bressman, Columbia-Presbyterian Medical Center, New York; W.J. Weiner and J. Sanchez-Ramos, University of Miami, Miami; J. Jankovic, Baylor College of Medicine, Houston; J.B. Penney, University of Michigan, Ann Arbor; A. Lang, Toronto Hospital, Toronto; M. Hoehn, St. Luke's Hospital, Denver; J. Tetrud, California Parkinson's Foundation, San Jose; J.D. Grimes, Ottawa Civic Hospital, Ottawa; R. Pfeiffer, University of Nebraska, Omaha; C. Shults and L. Thal, University of California, San Diego; S. Gauthier, Montreal General Hospital-McGill University, Montreal; L.I. Golbe, University of Medicine and Dentistry of New Jersey-Robert Wood Johnson Medical School, New Brunswick; J.S. Perlmutter, Washington University, St. Louis; H. Moses III and S.G. Reich, Johns Hopkins University, Baltimore; and H.I. Hurtig and M. Stern, Graduate Hospital and University of Pennsylvania, Philadelphia.

Site Coordinators -- R. Barter and B. Vetere-Overfield, Kansas City, Kans.; L. Gauger and T. Malapira, Tampa, Fla.; J. Dobson, Iowa City, Iowa; S. Atamian, M. Tennis, J.B. Cohen, G. Desclos, and E. Hoffman, Boston; L. Denio, S. Huber, T. Woike, K. Zoog, R. Mendell, and K. Dudte, Columbus, Ohio; J. Behr, I.F. Gardiner, Rochester, N.Y.; M. Lannon, Providence, R.I.; J. Carter and S. Northrup, Portland, Oreg.; B. Kanigan, Saskatoon, Sask.; M. Turk and E. Landow, Charlottesville, Va.; P. Schlick and K. Mistura, Detroit; V.S. Carroll and J.A. Thelen, Chicago; C. Demong, Calgary, Alta.; L. Winfield and C. Moskowitz, New York; A. Ingenito, C. Sheldon, and L. Cornelius, Miami; D. Heiberg and C. Dunne, Houston; J. Brady, Ann Arbor, Mich.; C. Kierans, L. Belle-Scantlebury, and J. Duff, Toronto; H. Weber, Denver; D. Savoini, P. Lewis, and S.J. Kutner, San Jose, Calif.; P. Gray, Ottawa; C. Glaeske and R. Hofman, Omaha, Neb.; M.M. Pay and D. Salmon, San Diego, Calif.; F. McFaul and D. Amyot, Montreal; M. Bergen, New Brunswick, N.J.; L. McGee-Minnich, St. Louis; P. O'Donnell, Baltimore; and S. Ferrise and K. Shallow, Philadelphia.

Coordination and Data Center -- University of Rochester Medical Center, Rochester, N.Y.: R.M. Pelusio (program manager); A. Rudolph (senior study coordinator); C. Miller (nurse clinician); M. Linsner, J. Connorton, J. Nusbaum, and C. Casaceli (analyst-programmers); C. Irvine, C. Orme, and G.J. Wixsom (information analysts); M. Schirazi, J. Sotack, and H. Randolph (data-control clerks); R. Nobel, D. Baker, D. LaDonna, M.E. Rothfuss, L. Doerr (deceased), L. Rumfola, and B. Kavanaugh (secretarial staff); and J. Wendel (CLINFO manager).

Biostatistics Center -- Department of Biostatistics, University of Rochester Medical Center, Rochester, N.Y.: C. Odoroff (deceased) and D. Oakes (chief biostatisticians); M. McDermott and S. Eberly (biostatisticians); S. Plumb (lead programmer); and A. Watts, L. Yorkey, A. Choi, and K. Gerwitz (analyst-programmers).

Pharmacy Center -- Strong Memorial Hospital, Rochester, N.Y.: P. Evans (chief pharmacist); and L. Dellapena and V. Singletary (pharmacy technicians).

Safety Monitoring Committee -- R. Herndon (chair, January 1, 1987, to June 30, 1988), Portland, Oreg.; P. Tariot (chair, July 1, 1988, to present), Rochester, N.Y.; and E. Bell, R.C. Griggs, and W.J. Hall, Rochester, N.Y.

Scientific Advisory Committee -- C.D. Marsden (chair), London; T.N. Chase, Bethesda, Md.; G. Cohen, J. Fleiss, and R. Mayeux, New York; L. Jacobs and A.J. Moss, Rochester, N.Y.; and E. Melamed, Tel Aviv, Israel.

Assay Standards Committee -- R. Roth (chair), New Haven, Conn.; M. Galloway, Detroit; I. Irwin, San Jose, Calif.; P. LeWitt, Detroit; and G. Vatassery, Minneapolis.

Neuropsychological Testing Committee -- P. Como (chair), Rochester, N.Y.; J. St. Cyr, Toronto; Y. Stern and J. Williams, New York; and R. Wilson, Chicago.

Monitoring Committee, National Institute of Neurological Disorders and Stroke -- E.M. Stadlan (chair), Bethesda, Md.; M. Alter, Philadelphia; K. Bergmann, New Hyde Park, N.Y.; J. Cedarbaum, White Plains, N.Y.; J. Ellenberg, Bethesda, Md.; and R. Kibler, Atlanta.

CSF Assay Center -- Lafayette Clinic, Wayne State University, Detroit: M.P. Galloway (director); M. Kaplan (deceased), R. Lodhi, M.J. Keegan, B. Matthews, and E.A. Novak.

Deprenyl Metabolites Assay Center -- Institute for Medical Research, San Jose, Calif.: I. Irwin (director).

Tocopherol Assay Center -- Our Lady of Mercy Medical Center, Bronx, N.Y.: E. Norkus (director).

Specimen Repository -- Department of Neurology, University of Rochester, Rochester, N.Y.: D. Flood (director), T. McNeill, N. Harary, and L. Koek.

Laboratory Surveillance Testing -- SciCor Laboratories, Indianapolis: R.L. Creveling (director).
